# 1-Methyl-3,3-bis­[(4-methyl­phen­yl)sulfan­yl]piperidin-2-one

**DOI:** 10.1107/S1600536811037111

**Published:** 2011-09-30

**Authors:** Julio Zukerman-Schpector, Paulo R. Olivato, Carlos R. Cerqueira Jr, Jean M. M. Santos, Seik Weng Ng, Edward R. T. Tiekink

**Affiliations:** a Department of Chemistry, Universidade Federal de São Carlos, 13565-905 São Carlos, SP, Brazil; b Chemistry Institute, Universidade de São Paulo, 05508-000 São Paulo-SP, Brazil; c Department of Chemistry, University of Malaya, 50603 Kuala Lumpur, Malaysia; d Chemistry Department, Faculty of Science, King Abdulaziz University, PO Box 80203 Jeddah, Saudi Arabia

## Abstract

The piperidone ring in the title compound, C_20_H_23_NOS_2_, has a half-chair distorted to a twisted-boat conformation [*Q*_T_ = 0.5200 (17) Å]. One of the S-bound benzene rings is almost perpendicular to the least-squares plane through the piperidone ring, whereas the other is not [dihedral angles = 75.28 (5) and 46.41 (5) Å, respectively]. In the crystal, the presence of C—H⋯O and C—H⋯π inter­actions leads to the formation of supra­molecular layers in the *ab* plane.

## Related literature

For background to β-thio­carbonyl compounds, see: Vinhato *et al.* (2011 ▶); Olivato *et al.* (2009 ▶). For related structures, see: Zukerman-Schpector *et al.* (2008 ▶, 2010 ▶). For ring conformational analysis, see: Cremer & Pople (1975 ▶). For the synthesis, see: Hashmat & McDermott (2002 ▶); Zoretic & Soja (1976 ▶).
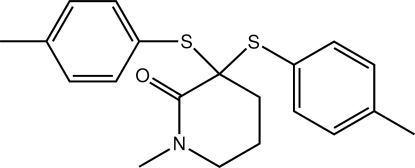


## Experimental

### 

#### Crystal data

C_20_H_23_NOS_2_
*M* *_r_* = 357.53
Monoclinic, 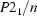

*a* = 7.8943 (1) Å
*b* = 9.8078 (2) Å
*c* = 23.9145 (4) Å
β = 92.803 (1)°
*V* = 1849.38 (5) Å^3^
*Z* = 4
Cu *K*α radiation
μ = 2.65 mm^−1^
*T* = 100 K
0.25 × 0.20 × 0.15 mm

#### Data collection

Agilent SuperNova Dual Cu at zero diffractometer with an Atlas detector
Absorption correction: multi-scan (*CrysAlis PRO*; Agilent, 2010 ▶) *T*_min_ = 0.558, *T*_max_ = 0.692
14169 measured reflections
3719 independent reflections
3465 reflections with *I* > 2σ(*I*)
*R*_int_ = 0.042

#### Refinement

*R*[*F* ^2^ > 2σ(*F* ^2^)] = 0.039
*wR*(*F* ^2^) = 0.107
*S* = 1.06
3719 reflections
220 parameters
H-atom parameters constrained
Δρ_max_ = 0.68 e Å^−3^
Δρ_min_ = −0.31 e Å^−3^

### 

Data collection: *CrysAlis PRO* (Agilent, 2010 ▶); cell refinement: *CrysAlis PRO*; data reduction: *CrysAlis PRO*; program(s) used to solve structure: *SIR92* (Altomare *et al.*, 1999 ▶); program(s) used to refine structure: *SHELXL97* (Sheldrick, 2008 ▶); molecular graphics: *ORTEP-3* (Farrugia, 1997 ▶) and *DIAMOND* (Brandenburg, 2006 ▶); software used to prepare material for publication: *publCIF* (Westrip, 2010 ▶).

## Supplementary Material

Crystal structure: contains datablock(s) global, I. DOI: 10.1107/S1600536811037111/hg5094sup1.cif

Structure factors: contains datablock(s) I. DOI: 10.1107/S1600536811037111/hg5094Isup2.hkl

Supplementary material file. DOI: 10.1107/S1600536811037111/hg5094Isup3.cml

Additional supplementary materials:  crystallographic information; 3D view; checkCIF report

## Figures and Tables

**Table 1:** Hydrogen-bond geometry (Å, °) *Cg*1 is the centroid of the C7–C12 ring.

*D*—H⋯*A*	*D*—H	H⋯*A*	*D*⋯*A*	*D*—H⋯*A*
C11—H11⋯O1^i^	0.95	2.37	3.294 (3)	166
C1—H1b⋯*Cg*1^ii^	0.98	2.84	3.624 (2)	137
C15—H15⋯*Cg*1^iii^	0.95	2.88	3.459 (2)	120

Symmetry codes: (i) 
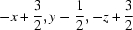
; (ii) 
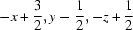
; (iii) 
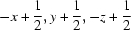
.

**Fig. 1.**
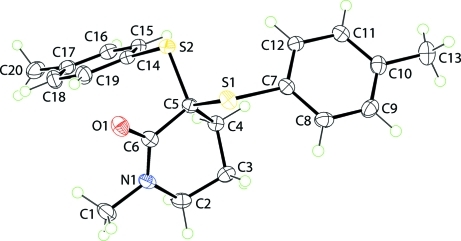
The molecular structure of (I) showing atom labelling scheme and displacement ellipsoids at the 50% probability level (arbitrary spheres for the H atoms).

**Fig. 2.**
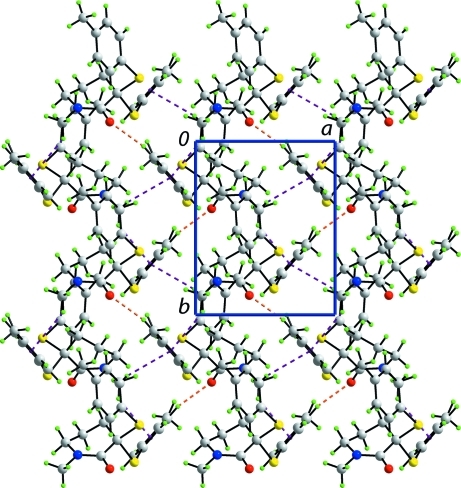
Supramolecular layer in the ab plane of (I) mediated by C—H···O and C—H···π interactions, shown as orange and purple dashed lines, respectively.

**Fig. 3.**
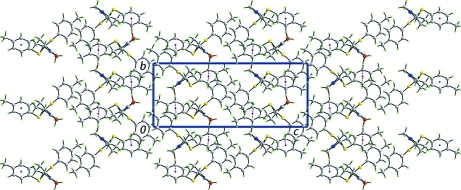
A view in projection down the a axis of the unit-cell contents of (I) showing the stacking of layers alog the c axis. The C—H···O and C—H···π interactions are shown as orange and purple dashed lines, respectively.

## supplementary crystallographic information

### Comment

As part of our on-going research on the conformational and electronic interactions in β-thio-carbonyl and β-bis-thio-carbonyl compounds, *e.g. N*,*N*-diethyl-2-[(4'-substituted)phelysulfonyl] acetamides, *N*-methoxy-*N*-methyl-2-[(4'-substituted) phenylthio]propanamides, 1-methyl-3-phenylsulfonyl-2-piperidone and 3,3-bis[(4-chlorophenyl)sulfanyl]-1-methyl-2-piperidone, utilizing spectroscopic, theoretical and X-ray diffraction methods (Vinhato, *et al.* 2011; Olivato *et al.*, 2009; Zukerman-Schpector *et al.* 2008, 2010), the title compound, (I), was synthesized and its crystal structure determined.

In (I), Fig. 1, the piperidone ring has a distorted half-chair conformation with the C3 atom lying 0.687 (2) Å out of the plane defined by the other five atoms (r.m.s. deviation = 0.0833 Å). The ring puckering parameters are: q_2_ = 0.4309 (17) Å, q_3_ = 0.2909 (17) Å, QT = 0.5200 (17) Å, φ_2_ = 145.6 (2) ° (Cremer & Pople, 1975). The S2-bound benzene ring is orientated to be almost perpendicular to the plane through the piperidone ring [dihedral angle = 75.28 (5) °]. The S1-bond benzene ring is somewhat splayed with respect to the other rings, forming dihedral angles of 46.41 (5) and 59.02 (5) ° with those through the piperidone and S2-bound benzene rings, respectively.

The crystal packing of (I), Table 1, is sustained by C—H···O and C—H···π interactions that lead to the formation of supramolecular layers in the *ab* plane, Fig. 1. The S1-benzene accepts to C—H···π contacts. Layers stack along the *c* axis as illustrated in Fig.3.

### Experimental

Firstly, 4-methylthiophenol (5.0 g, 40 mmol) was reacted with bromine (1.1 ml, 20 mmol) in dichloromethane (250 ml) on hydrated silica gel support (25 g of SiO_2_ and 12 ml of water) to give 4-methylphenyl disulfide (4.1 g, yield = 83%). A white solid was obtained after filtration and evaporation without further purification (Hashmat & McDermott, 2002). 1-Methyl-2-piperidinone (1.9 g, 17 mmol) was added drop-wise to a cooled (195 K) solution of hexamethylphosphoramide (HMPA) (3.1 ml, 17 mmol), diisopropylamine (2.6 ml, 17 mmol) and butyllithium (13.5 ml, 17 mmol) in THF (60 ml). After 20 minutes, 4-methylphenyl disulfide (4.1 g, 17 mmol) dissolved in THF (10 ml) was added drop-wise to the enolate solution (Zoretic & Soja, 1976). After the mixture was stirred for 4 h at 195 K, water (80 ml) was added at room temperature and extraction with chloroform was performed. The organic layer was dried over anhydrous sodium sulfate. After evaporation of solvent, a crude solid was obtained. Purification through flash chromatography with a solution of hexane and ethyl acetate in a 7:3 ratio give the pure product (3.3 g, yield = 56%). Suitable crystals for X-ray analysis were obtained by vapour diffusion of *n*-hexane into a chloroform solution of (I) held at 283 K; m.p. 392–393 K. IR (cm^-1^): ν(C=O) 1662. NMR (CDCl_3_, p.p.m.): δ 1.88–1.93 (2*H*, m), 1.96–1.99 (2*H*, m), 2.38 (6*H*, s), 2.93 (3*H*, s), 3.163.18 (2*H*, t, J = 6.1 Hz), 7.15–7.17 (4*H*, d, J = 7.8 Hz,Aryl-H), 7.52–7.54 (4*H*, m, Aryl-H). Analysis found: C 67.22, H 6.45, N 3.95%. C_20_H_23_ONS_2_ requires: C 67.19, H 6.48, N 3.92%.

### Refinement

The H atoms were geometrically placed (C–H = 0.95–0.99 Å) and refined as riding with *U_iso_*(H) = 1.2–1.5*U_eq_*(C).

### Crystal data

**Table:**

C_20_H_23_NOS_2_	*F*(000) = 760
*M**_r_* = 357.53	*D*_x_ = 1.284 Mg m^−^^3^
Monoclinic, *P*2_1_/*n*	Cu *K*α radiation, λ = 1.54184 Å
Hall symbol: -P 2yn	Cell parameters from 8545 reflections
*a* = 7.8943 (1) Å	θ = 3.7–74.2°
*b* = 9.8078 (2) Å	µ = 2.65 mm^−^^1^
*c* = 23.9145 (4) Å	*T* = 100 K
β = 92.803 (1)°	Block, colourless
*V* = 1849.38 (5) Å^3^	0.25 × 0.20 × 0.15 mm
*Z* = 4	

### Data collection

**Table:**

Agilent SuperNova Dual Cu at zero diffractometer with an Atlas detector	3719 independent reflections
Radiation source: fine-focus sealed tube	3465 reflections with *I* > 2σ(*I*)
graphite	*R*_int_ = 0.042
Detector resolution: 10.4041 pixels mm^-1^	θ_max_ = 74.4°, θ_min_ = 3.7°
ω scans	*h* = −7→9
Absorption correction: multi-scan (*CrysAlis PRO*; Agilent, 2010)	*k* = −11→12
*T*_min_ = 0.558, *T*_max_ = 0.692	*l* = −29→29
14169 measured reflections	

### Refinement

**Table:**

Refinement on *F*^2^	Primary atom site location: structure-invariant direct methods
Least-squares matrix: full	Secondary atom site location: difference Fourier map
*R*[*F*^2^ > 2σ(*F*^2^)] = 0.039	Hydrogen site location: inferred from neighbouring sites
*wR*(*F*^2^) = 0.107	H-atom parameters constrained
*S* = 1.06	*w* = 1/[σ^2^(*F*_o_^2^) + (0.0608*P*)^2^ + 0.7727*P*] where *P* = (*F*_o_^2^ + 2*F*_c_^2^)/3
3719 reflections	(Δ/σ)_max_ < 0.001
220 parameters	Δρ_max_ = 0.68 e Å^−^^3^
0 restraints	Δρ_min_ = −0.31 e Å^−^^3^

### Special details

**Table:**

Geometry. All s.u.'s (except the s.u. in the dihedral angle between two l.s. planes) are estimated using the full covariance matrix. The cell s.u.'s are taken into account individually in the estimation of s.u.'s in distances, angles and torsion angles; correlations between s.u.'s in cell parameters are only used when they are defined by crystal symmetry. An approximate (isotropic) treatment of cell s.u.'s is used for estimating s.u.'s involving l.s. planes.
Refinement. Refinement of *F*^2^ against ALL reflections. The weighted *R*-factor *wR* and goodness of fit *S* are based on *F*^2^, conventional *R*-factors *R* are based on *F*, with *F* set to zero for negative *F*^2^. The threshold expression of *F*^2^ > σ(*F*^2^) is used only for calculating *R*-factors(gt) *etc*. and is not relevant to the choice of reflections for refinement. *R*-factors based on *F*^2^ are statistically about twice as large as those based on *F*, and *R*- factors based on ALL data will be even larger.

### Fractional atomic coordinates and isotropic or equivalent isotropic displacement parameters (Å^2^)

**Table:**

	*x*	*y*	*z*	*U*_iso_*/*U*_eq_	
S1	0.56118 (5)	0.85032 (4)	0.742407 (16)	0.02168 (13)	
S2	0.59643 (5)	0.63592 (4)	0.658865 (16)	0.02231 (13)	
N1	0.15516 (17)	0.80815 (15)	0.66875 (6)	0.0232 (3)	
O1	0.38510 (15)	0.88872 (12)	0.62753 (5)	0.0266 (3)	
C1	0.0471 (2)	0.8882 (2)	0.62973 (8)	0.0307 (4)	
H1A	0.0871	0.8783	0.5918	0.046*	
H1B	0.0517	0.9844	0.6407	0.046*	
H1C	−0.0701	0.8554	0.6305	0.046*	
C2	0.0687 (2)	0.73621 (18)	0.71307 (7)	0.0265 (3)	
H2A	0.0091	0.6556	0.6968	0.032*	
H2B	−0.0174	0.7973	0.7284	0.032*	
C3	0.1907 (2)	0.69027 (17)	0.76010 (7)	0.0229 (3)	
H3A	0.1333	0.6258	0.7848	0.028*	
H3B	0.2293	0.7698	0.7828	0.028*	
C4	0.3418 (2)	0.62117 (16)	0.73509 (6)	0.0201 (3)	
H4A	0.3021	0.5441	0.7112	0.024*	
H4B	0.4182	0.5844	0.7655	0.024*	
C5	0.43854 (18)	0.72223 (16)	0.70031 (6)	0.0193 (3)	
C6	0.32331 (19)	0.81387 (16)	0.66234 (6)	0.0204 (3)	
C7	0.62175 (19)	0.76581 (16)	0.80585 (6)	0.0194 (3)	
C8	0.5588 (2)	0.81362 (17)	0.85544 (7)	0.0240 (3)	
H8	0.4773	0.8849	0.8546	0.029*	
C9	0.6155 (2)	0.75683 (18)	0.90622 (7)	0.0259 (4)	
H9	0.5729	0.7907	0.9400	0.031*	
C10	0.7334 (2)	0.65141 (17)	0.90856 (7)	0.0241 (3)	
C11	0.7935 (2)	0.60218 (16)	0.85848 (7)	0.0220 (3)	
H11	0.8727	0.5292	0.8592	0.026*	
C12	0.73859 (19)	0.65906 (16)	0.80755 (6)	0.0197 (3)	
H12	0.7808	0.6251	0.7737	0.024*	
C13	0.7979 (3)	0.5920 (2)	0.96396 (7)	0.0344 (4)	
H13	0.7035	0.5831	0.9889	0.052*	
H13B	0.8844	0.6524	0.9812	0.052*	
H13C	0.8474	0.5020	0.9577	0.052*	
C14	0.46802 (19)	0.53540 (17)	0.61152 (6)	0.0220 (3)	
C15	0.4551 (2)	0.39502 (18)	0.61902 (7)	0.0242 (3)	
H15	0.5083	0.3533	0.6512	0.029*	
C16	0.3648 (2)	0.31557 (19)	0.57978 (7)	0.0273 (4)	
H16	0.3568	0.2199	0.5854	0.033*	
C17	0.2859 (2)	0.37422 (19)	0.53241 (7)	0.0276 (4)	
C18	0.2970 (2)	0.5147 (2)	0.52567 (7)	0.0318 (4)	
H18	0.2420	0.5563	0.4938	0.038*	
C19	0.3866 (2)	0.59541 (19)	0.56450 (7)	0.0288 (4)	
H19	0.3926	0.6912	0.5591	0.035*	
C20	0.1961 (2)	0.2880 (2)	0.48744 (8)	0.0356 (4)	
H20A	0.0953	0.3364	0.4724	0.053*	
H20B	0.1621	0.2010	0.5036	0.053*	
H20C	0.2730	0.2709	0.4572	0.053*	

### Atomic displacement parameters (Å^2^)

**Table:**

	*U*^11^	*U*^22^	*U*^33^	*U*^12^	*U*^13^	*U*^23^
S1	0.0222 (2)	0.0165 (2)	0.0259 (2)	−0.00289 (13)	−0.00351 (15)	0.00200 (13)
S2	0.0170 (2)	0.0272 (2)	0.0228 (2)	0.00184 (14)	0.00158 (14)	−0.00095 (14)
N1	0.0186 (6)	0.0239 (7)	0.0269 (7)	0.0039 (5)	−0.0015 (5)	−0.0002 (5)
O1	0.0270 (6)	0.0237 (6)	0.0289 (6)	−0.0027 (5)	−0.0023 (5)	0.0067 (5)
C1	0.0246 (9)	0.0338 (10)	0.0329 (9)	0.0076 (7)	−0.0077 (7)	−0.0009 (7)
C2	0.0188 (8)	0.0266 (9)	0.0342 (9)	−0.0007 (6)	0.0038 (6)	−0.0022 (7)
C3	0.0221 (8)	0.0212 (8)	0.0259 (8)	−0.0039 (6)	0.0055 (6)	−0.0023 (6)
C4	0.0201 (7)	0.0169 (7)	0.0233 (8)	−0.0018 (6)	0.0011 (6)	−0.0005 (6)
C5	0.0174 (7)	0.0184 (7)	0.0221 (7)	−0.0003 (6)	0.0005 (5)	0.0001 (6)
C6	0.0209 (7)	0.0154 (7)	0.0246 (8)	−0.0002 (6)	−0.0018 (6)	−0.0017 (6)
C7	0.0178 (7)	0.0176 (7)	0.0225 (7)	−0.0032 (6)	−0.0016 (5)	−0.0002 (6)
C8	0.0201 (8)	0.0225 (8)	0.0295 (8)	0.0011 (6)	0.0012 (6)	−0.0055 (6)
C9	0.0253 (8)	0.0297 (9)	0.0231 (8)	−0.0047 (7)	0.0043 (6)	−0.0069 (6)
C10	0.0248 (8)	0.0252 (8)	0.0219 (8)	−0.0079 (6)	−0.0012 (6)	−0.0001 (6)
C11	0.0208 (7)	0.0190 (8)	0.0260 (8)	−0.0013 (6)	−0.0013 (6)	0.0001 (6)
C12	0.0180 (7)	0.0190 (8)	0.0221 (7)	−0.0017 (6)	0.0017 (6)	−0.0031 (5)
C13	0.0435 (11)	0.0347 (10)	0.0244 (9)	−0.0040 (8)	−0.0040 (7)	0.0023 (7)
C14	0.0193 (7)	0.0265 (8)	0.0204 (7)	0.0044 (6)	0.0032 (5)	−0.0016 (6)
C15	0.0234 (8)	0.0265 (8)	0.0226 (8)	0.0041 (6)	0.0027 (6)	0.0016 (6)
C16	0.0270 (8)	0.0256 (9)	0.0297 (8)	0.0005 (7)	0.0062 (6)	−0.0023 (7)
C17	0.0216 (8)	0.0363 (10)	0.0251 (8)	0.0026 (7)	0.0029 (6)	−0.0070 (7)
C18	0.0353 (9)	0.0364 (10)	0.0232 (8)	0.0098 (8)	−0.0046 (7)	−0.0013 (7)
C19	0.0346 (9)	0.0267 (9)	0.0250 (8)	0.0059 (7)	−0.0008 (7)	0.0009 (7)
C20	0.0312 (9)	0.0441 (11)	0.0316 (9)	−0.0026 (8)	0.0034 (7)	−0.0116 (8)

### Geometric parameters (Å, °)

**Table:**

S1—C7	1.7738 (16)	C9—C10	1.390 (3)
S1—C5	1.8531 (16)	C9—H9	0.9500
S2—C14	1.7802 (17)	C10—C11	1.396 (2)
S2—C5	1.8366 (16)	C10—C13	1.513 (2)
N1—C6	1.345 (2)	C11—C12	1.390 (2)
N1—C1	1.462 (2)	C11—H11	0.9500
N1—C2	1.469 (2)	C12—H12	0.9500
O1—C6	1.229 (2)	C13—H13	0.9800
C1—H1A	0.9800	C13—H13B	0.9800
C1—H1B	0.9800	C13—H13C	0.9800
C1—H1C	0.9800	C14—C15	1.393 (2)
C2—C3	1.513 (2)	C14—C19	1.398 (2)
C2—H2A	0.9900	C15—C16	1.390 (2)
C2—H2B	0.9900	C15—H15	0.9500
C3—C4	1.520 (2)	C16—C17	1.390 (2)
C3—H3A	0.9900	C16—H16	0.9500
C3—H3B	0.9900	C17—C18	1.391 (3)
C4—C5	1.523 (2)	C17—C20	1.516 (2)
C4—H4A	0.9900	C18—C19	1.387 (3)
C4—H4B	0.9900	C18—H18	0.9500
C5—C6	1.542 (2)	C19—H19	0.9500
C7—C8	1.389 (2)	C20—H20A	0.9800
C7—C12	1.395 (2)	C20—H20B	0.9800
C8—C9	1.390 (2)	C20—H20C	0.9800
C8—H8	0.9500		
			
C7—S1—C5	105.06 (7)	C9—C8—H8	120.1
C14—S2—C5	102.60 (7)	C8—C9—C10	121.29 (15)
C6—N1—C1	116.97 (14)	C8—C9—H9	119.4
C6—N1—C2	126.74 (14)	C10—C9—H9	119.4
C1—N1—C2	116.19 (14)	C9—C10—C11	118.54 (15)
N1—C1—H1A	109.5	C9—C10—C13	121.17 (16)
N1—C1—H1B	109.5	C11—C10—C13	120.28 (16)
H1A—C1—H1B	109.5	C12—C11—C10	120.56 (16)
N1—C1—H1C	109.5	C12—C11—H11	119.7
H1A—C1—H1C	109.5	C10—C11—H11	119.7
H1B—C1—H1C	109.5	C11—C12—C7	120.31 (15)
N1—C2—C3	112.24 (13)	C11—C12—H12	119.8
N1—C2—H2A	109.2	C7—C12—H12	119.8
C3—C2—H2A	109.2	C10—C13—H13	109.5
N1—C2—H2B	109.2	C10—C13—H13B	109.5
C3—C2—H2B	109.2	H13—C13—H13B	109.5
H2A—C2—H2B	107.9	C10—C13—H13C	109.5
C2—C3—C4	108.87 (13)	H13—C13—H13C	109.5
C2—C3—H3A	109.9	H13B—C13—H13C	109.5
C4—C3—H3A	109.9	C15—C14—C19	119.09 (15)
C2—C3—H3B	109.9	C15—C14—S2	120.57 (12)
C4—C3—H3B	109.9	C19—C14—S2	120.24 (13)
H3A—C3—H3B	108.3	C16—C15—C14	120.38 (15)
C3—C4—C5	110.42 (13)	C16—C15—H15	119.8
C3—C4—H4A	109.6	C14—C15—H15	119.8
C5—C4—H4A	109.6	C15—C16—C17	120.85 (17)
C3—C4—H4B	109.6	C15—C16—H16	119.6
C5—C4—H4B	109.6	C17—C16—H16	119.6
H4A—C4—H4B	108.1	C16—C17—C18	118.45 (16)
C4—C5—C6	113.82 (12)	C16—C17—C20	121.49 (17)
C4—C5—S2	111.49 (11)	C18—C17—C20	120.01 (17)
C6—C5—S2	110.31 (10)	C19—C18—C17	121.37 (16)
C4—C5—S1	114.02 (10)	C19—C18—H18	119.3
C6—C5—S1	101.67 (10)	C17—C18—H18	119.3
S2—C5—S1	104.79 (7)	C18—C19—C14	119.84 (17)
O1—C6—N1	121.93 (14)	C18—C19—H19	120.1
O1—C6—C5	120.32 (14)	C14—C19—H19	120.1
N1—C6—C5	117.75 (13)	C17—C20—H20A	109.5
C8—C7—C12	119.48 (15)	C17—C20—H20B	109.5
C8—C7—S1	118.66 (12)	H20A—C20—H20B	109.5
C12—C7—S1	121.73 (12)	C17—C20—H20C	109.5
C7—C8—C9	119.81 (16)	H20A—C20—H20C	109.5
C7—C8—H8	120.1	H20B—C20—H20C	109.5
			
C6—N1—C2—C3	12.2 (2)	C5—S1—C7—C12	68.39 (14)
C1—N1—C2—C3	−163.84 (14)	C12—C7—C8—C9	1.4 (2)
N1—C2—C3—C4	−47.35 (18)	S1—C7—C8—C9	−174.50 (12)
C2—C3—C4—C5	63.55 (16)	C7—C8—C9—C10	−0.7 (2)
C3—C4—C5—C6	−43.61 (17)	C8—C9—C10—C11	−0.5 (2)
C3—C4—C5—S2	−169.18 (10)	C8—C9—C10—C13	178.60 (16)
C3—C4—C5—S1	72.41 (14)	C9—C10—C11—C12	1.1 (2)
C14—S2—C5—C4	65.40 (12)	C13—C10—C11—C12	−178.10 (15)
C14—S2—C5—C6	−62.08 (12)	C10—C11—C12—C7	−0.3 (2)
C14—S2—C5—S1	−170.79 (8)	C8—C7—C12—C11	−0.9 (2)
C7—S1—C5—C4	31.05 (13)	S1—C7—C12—C11	174.87 (12)
C7—S1—C5—C6	153.97 (10)	C5—S2—C14—C15	−105.11 (14)
C7—S1—C5—S2	−91.12 (8)	C5—S2—C14—C19	78.55 (14)
C1—N1—C6—O1	4.1 (2)	C19—C14—C15—C16	1.1 (2)
C2—N1—C6—O1	−171.93 (15)	S2—C14—C15—C16	−175.31 (12)
C1—N1—C6—C5	−175.70 (14)	C14—C15—C16—C17	0.0 (2)
C2—N1—C6—C5	8.2 (2)	C15—C16—C17—C18	−1.1 (3)
C4—C5—C6—O1	−171.64 (14)	C15—C16—C17—C20	176.18 (16)
S2—C5—C6—O1	−45.45 (17)	C16—C17—C18—C19	1.1 (3)
S1—C5—C6—O1	65.30 (16)	C20—C17—C18—C19	−176.26 (16)
C4—C5—C6—N1	8.2 (2)	C17—C18—C19—C14	0.0 (3)
S2—C5—C6—N1	134.37 (13)	C15—C14—C19—C18	−1.1 (2)
S1—C5—C6—N1	−114.88 (13)	S2—C14—C19—C18	175.28 (14)
C5—S1—C7—C8	−115.80 (13)		

### Hydrogen-bond geometry (Å, °)

**Table:**

Cg1 is the centroid of the C7–C12 ring.

**Table:**

*D*—H···*A*	*D*—H	H···*A*	*D*···*A*	*D*—H···*A*
C11—H11···O1^i^	0.95	2.37	3.294 (3)	166
C1—H1b···Cg1^ii^	0.98	2.84	3.624 (2)	137
C15—H15···Cg1^iii^	0.95	2.88	3.459 (2)	120

Symmetry codes: (i) −*x*+3/2, *y*−1/2, −*z*+3/2; (ii) −*x*+3/2, *y*−1/2, −*z*+1/2; (iii) −*x*+1/2, *y*+1/2, −*z*+1/2.
